# Skin Barrier– and Immune Response–Related Biomarkers of Solar UVR Exposure Comparing Indoor and Outdoor Workers

**DOI:** 10.1016/j.xjidi.2024.100280

**Published:** 2024-04-16

**Authors:** Florentine L. de Boer, Henk F. van der Molen, Jen-Hung Wang, Ellen Raun, Jorge Pereda, Edwin En-Te Hwu, Ivone Jakasa, Sandrine Dubrac, Thomas Rustemeyer, Sanja Kezic

**Affiliations:** 1 Amsterdam Public Health Research Institute, Department of Public and Occupational Health, Amsterdam University Medical Center, University of Amsterdam, Amsterdam, The Netherlands; 2 Department of Health Technology, Technical University of Denmark, Kongens Lyngby, Denmark; 3 Laboratory for Analytical Chemistry, Department of Chemistry and Biochemistry, Faculty of Food Technology and Biotechnology, University of Zagreb, Zagreb, Croatia; 4 Epidermal Biology Laboratory, Department of Dermatology, Venereology and Allergology, Medical University of Innsbruck, Innsbruck, Austria; 5 Dermato-Allergology and Occupational Dermatology, Amsterdam University Medical Centers, Noord-Holland, The Netherlands

**Keywords:** Biomarkers, Occupational UVR exposure, Stratum corneum, UVR

## Abstract

Outdoor workers have increased risk of developing keratinocyte cancer due to accumulated skin damage resulting from chronic and excessive exposure to UVR. This study aims to identify potential noninvasive biomarkers to assess chronic UVR exposure. We analyzed stratum corneum biomarkers collected from 2 skin locations and 2 occupational groups with contrasting solar UVR exposure: the forehead and retroauricular skin among outdoor workers and indoor workers. Using a linear mixed model adjusting for age and skin phototype, we compared biomarkers between both skin sites in indoor and outdoor workers. We measured markers of the immune response and skin barrier, including cytokines, GFs, 15-hydroxyeicosatetraenoic acid, cis- and *trans*-urocanic acid, and corneocyte topography, indicated by circular nano objects. Differences between the 2 skin sites were found for cis-urocanic acid, total urocanic acid, IL-1α, IL-1RA, IL-1RA/IL-1α, IL-18, 15-hydroxyeicosatetraenoic acid, CCL4, and circular nano objects. The levels of cis-urocanic acid and CCL4 also differed between indoor and outdoor workers. These findings underscore changes in both immune response and skin barrier induced by UVR. They indicate the potential utility of stratum corneum biomarkers in detecting both chronic UVR exposure in occupational setting and aiding in the development of preventive measures.

## Introduction

Keratinocyte cancer, comprising basal cell carcinoma and squamous cell carcinoma, is the most common cancer type (Fitzmaurice et al., 2019), primarily caused by UVR. Outdoor workers (OWs) such as construction or agricultural workers, who spend more time outdoors than indoor workers (IWs), are at higher risk to develop keratinocyte cancer (Trakatelli et al, 2016). Among OWs, the risk of developing squamous cell carcinoma is increased by 77% and by 43% for basal cell carcinoma compared with that among IWs (Bauer et al, 2011; Schmitt et al, 2011). Furthermore, OWs have up to 3-fold higher incidence of keratinocyte cancer than the general population (John et al, 2016). Recent assessments using personal dosimeters have revealed considerably higher levels of UVR exposure in OWs than in the general population (John et al, 2021; Wittlich et al, 2020), hereby explaining their increased risk of developing keratinocyte cancer.

Keratinocyte cancer has profound impact on the QOL owing to its chronicity and frequent recurrence. Patients often need to undergo repeated surgery on highly visible, sun-exposed areas (eg, head, ears, neck, and hands) (Răducu et al, 2020). The high burden of chronic UVR exposure for both the individual and the healthcare system can be largely avoided if targeted and personalized prevention measures are utilized (Kornek and Augustin, 2013). In occupational settings, various interventions aiming at reducing UVR exposure have been proposed (John et al, 2016), but their effectiveness remains insufficiently investigated, mainly owing to the lack of objective outcomes. Keratinocyte cancer has a long latency period of 20–30 years (Nanz et al, 2024) and typically manifest at an older age (around age 70 years on average [Kwiatkowska et al, 2021]), making it unsuitable for short-term evaluation of preventive measures. Current assessments rely on self-reported questionnaires regarding perceived risk awareness and compliance with protective measures, which are prone to self-report bias.

Biomarkers associated with UVR exposure could provide an objective method for assessing the UVR exposure as well as the impact of prevention measures. The selection of biomarkers was based on existing literature demonstrating their detectability in stratum corneum (SC) tape strips as well as their relevance to UVR exposure (Hulshof et al, 2019; Keurentjes et al, 2022).

Recent studies identified various SC biomarkers indicative of UVR exposure. However, these studies were carried out in young, healthy volunteers exposed to relatively low UVR dose and for a short time period (Keurentjes et al, 2022, 2020b). In this study, we investigate whether some of these biomarkers are applicable for assessing chronic solar exposure, which is more relevant to real-life situations. The noninvasive and simple collection of SC samples is of importance for their implementation in occupational settings. As a skin barrier biomarker, we measured corneocyte surface topography, indicated by a number of circular nano objects (CNOs) on the corneocyte surface. A recent study using atomic force microscopy (AFM) has demonstrated the usefulness of corneocyte surface topography as a valuable tool for detecting actinic damage in the adjacent, apparently normal skin near actinic keratosis lesion (Keurentjes et al, 2020a). cis- and *trans*-urocanic acid (UCA) were measured as a biomarker for UVR exposure and as a biomarker of skin barrier (Keurentjes et al, 2022; Rawlings and Harding, 2004). Among immunological biomarkers, we included cytokines of different signature, GFs, matrix metallopeptidase, and an anti-inflammatory eicosanoid (15-hydroxyeicosatetraenoic acid [15-HETE]).

To investigate variations in SC biomarker levels associated with different UVR exposure, we compared the levels of biomarkers in the forehead skin with the levels of biomarkers in retroauricular skin in IWs and OWs by means of noninvasive tape strip samples. Our initial assumption is that the forehead skin site and OWs are more exposed to UVR than retroauricular skin site and IWs.

## Results

### Demographic characteristics

This observational study comprised of 29 healthy male OWs, with a mean age of 46 years (range = 18–64 years), and 31 healthy IWs with a mean age of 46 years (range = 21–57 years). The OWs were recruited from 2 construction companies as a result of a convenience sampling method. The IWs were recruited from the same construction companies and were employees who mainly worked at the office. The IWs did not work outdoors for 4 hours or more, whereas the OWs did. All participants were classified according to Fitzpatrick phototypes I–VI (Fitzpatrick, 1988). Participants with Fitzpatrick phototype I have very light skin, hair, and eyes and get sunburned easily, whereas people with Fitzpatrick phototype VI have very pigmented skin and usually do not get sunburned. Among OWs and IWs, skin Fitzpatrick phototypes II and III were the most frequent. OWs had a higher proportion (66.7%) of skin phototype III than IWs (50%) (Table 1).

**Table 1:** Demographic Characteristics: Age, Work History, and Fitzpatrick Skin Phototype

Characteristic	IWs (n = 31)	OWs (n = 29)
Age, y, median (IQR)	47 (36–56)	52 (32–60)
Work history, y, median (IQR)	20 (15–28)	26 (13–35)
Skin phototype II, n (%)	8 (28.57)	7 (23.33)
Skin phototype III, n (%)	14 (50.00)	20 (66.67)
Skin phototype IV, n (%)	3 (10.71)	2 (6.67)
Skin phototype V, n (%)	1 (3.57)	0 (0.00)
Skin phototype VI, n (%)	2 (7.14)	1 (3.33)

Abbreviations: IQR, interquartile range; IW, indoor worker; OW, outdoor worker.

### Comparing 2 skin sites (forehead and retroauricular skin)

**Figure 1:**
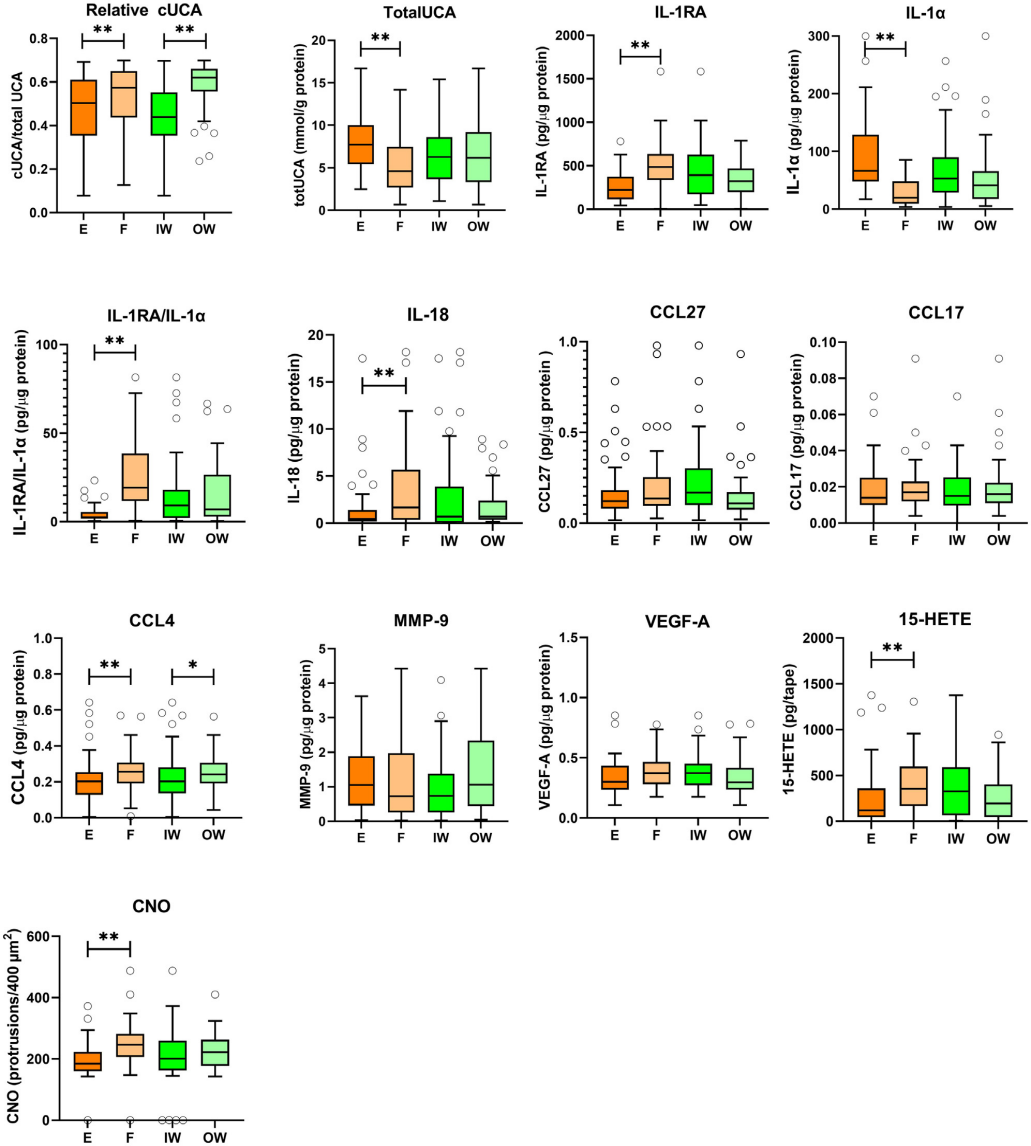
**SC immunological biomarker levels on skin sites F and E and in IWs and OWs.** These include the relative amount of cUCA (n = 110), total UCA (n = 115), IL-1RA (n = 118), IL-1α (n = 115), IL-1RA/IL-1α (n = 107), IL-18 (n = 101), CCL27 (n = 106), CCL17 (n = 111), CCL4 (n = 115), MMP-9 (n = 104), VEGF-A (n = 115), 15-HETE (n = 112), and CNO (n = 95). Data are given as median and interquartile ranges. Differences between the 2 skin sites and between IWs and OWs were tested using a linear mixed model. A Benjamini–Hochberg test was performed to correct for multiple testing (protrusions/400 μm^2^). ∗*P* < .05, ∗∗*P* < .01, ∗∗∗*P* < .001, and ∗∗∗∗*P* < .0001. E denotes retroauricular skin, and F denotes forehead skin. 15-HETE, 15-hydroxyeicosatetraenoic acid; CNO, circular nano object; cUCA, cis-urocanic acid; IW, indoor worker; MMP-9, matrix metalloproteinase 9; OW, outdoor worker; SC, stratum corneum; UCA, urocanic acid.

Significant differences between forehead skin and retroauricular skin sites were observed in 8 of 13 investigated biomarkers (Figure 1 and Table 2). An interaction effect between skin site and occupational group was observed for relative amount of cis*-*UCA (cis-UCA/total UCA). No significant effect of age or phototype were detected for biomarkers, except for phototype with regard to relative cis-UCA.

**Table 2:** Stratum Corneum Biomarker Levels on the Forehead and Behind the Ear Measured in IWs and OWs

Biomarker (pg/μg Protein)	E (Median with IQR)	F (Median with IQR)	Adjusted *P*-value^1^	IW (Median with IQR)	OW (Median with IQR)	Adjusted *P*-value	IW/OW × E/F^2^
cUCA^3^	0.504 (0.354–0.610)	0.574 (0.437–0.650)	.002	0.439 (0.354–0.553)	0.620 (0.557–0.660)	.002	0.024
Total UCA	7.733 (5.437–10.010)	4.612 (2.677–7.481)	.002	6.270 (3.658-8.608)	6.165 (3.300–9.180)	.317	0.444
IL-1RA	222.2 (113.9–374.100)	486.2 (336.700–636.600)	.002	392.200 (172.100–628.700)	322.900 (197.700–469.800)	.306	0.118
IL-1α	66.41 (47.870–128.800)	19.510 (9.474–48.130)	.002	53.110 (28.610–89.880)	41.260 (17.350–65.950)	.309	0.997
IL-1RA/IL-1α	2.516 (1.622–5.456)	19.230 (11.690–38.440)	.002	9.085 (2.162–18.030)	6.941 (2.893–26.580)	.589	0.206
IL-18	0.438 (0.181–1.393)	1.666 (0.341–5.672)	.002	0.691 (0.033–3.885)	0.670 (0.339–2.388)	.172	0.171
CCL27	0.121 (0.080–0.181)	0.136 (0.095–0.254)	.299	0.168 (0.010–0.302)	0.109 (0.074–0.171)	.306	0.421
CCL17	0.014 (0.010–0.025)	0.017 (0.012–0.023)	.551	0.015 (0.010–0.025)	0.016 (0.011–0.022)	.299	0.773
CCL4	0.203 (0.128–0.254)	0.256 (0.191–0.307)	.006	0.203 (0.136–0.281)	0.242 (0.191–0.306)	.028	0.445
MMP-9	1.056 (0.457–1.884)	0.726 (0.261–1.979)	.306	0.739 (0.260–1.375)	1.065 (0.443–2.340)	.396	1.000
VEGF-A	0.300 (0.235–0.434)	0.372 (0.280–0.466)	.111	0.372 (0.272–0.452)	0.297 (0.236–0.416)	.309	0.504
15-HETE (pg/tape)	118.700 (45.580–359.200)	354.500 (167.200–597.300)	.002	327.400 (66.760–591.600)	195.400 (48.010–402.600)	.266	0.141
CNO (protrusions/400 μm^2^)	184.500 (160.100–222.900)	246.100 (206.100–282.000)	.002	200.600 (162.800–259.300)	222.300 (177.300–262.600)	.523	0.570

Abbreviations: 15-HETE, 15-hydroxyeicosatetraenoic acid; CNO, circular nano object; cUCA, cis-urocanic acid; IQR, interquartile range; IW, indoor worker; MMP-9, matrix metalloproteinase 9; OW, outdoor worker; SC, stratum corneum.

Data are given as median and interquartile ranges. E denotes retroauricular skin, and F denotes forehead skins.

1 Differences between the 2 skin sites and between IWs and OWs were tested by the linear regression mixed model. *P*-values were adjusted for multiple testing using Benjamini–Hochberg procedure.

2 Combined interaction effect between IWs and OWs and skin locations F and E.

3 Relative amount of cUCA (cUCA/total UCA).

Among immunological biomarkers, significant differences between the 2 skin sites were found for IL-1RA, IL-1α, IL-1RA/IL-1α, IL-18, CCL4, and 15-HETE. Except for IL-1α, which showed lower levels in the forehead skin, all other biomarkers demonstrated higher levels in the forehead than in the retroauricular skin location. No significant differences between the 2 skin sites were detected for CCL27, matrix metalloproteinase 9, VEGFA, and CCL17 (Figure 1 and Table 2).

**Figure 2:**
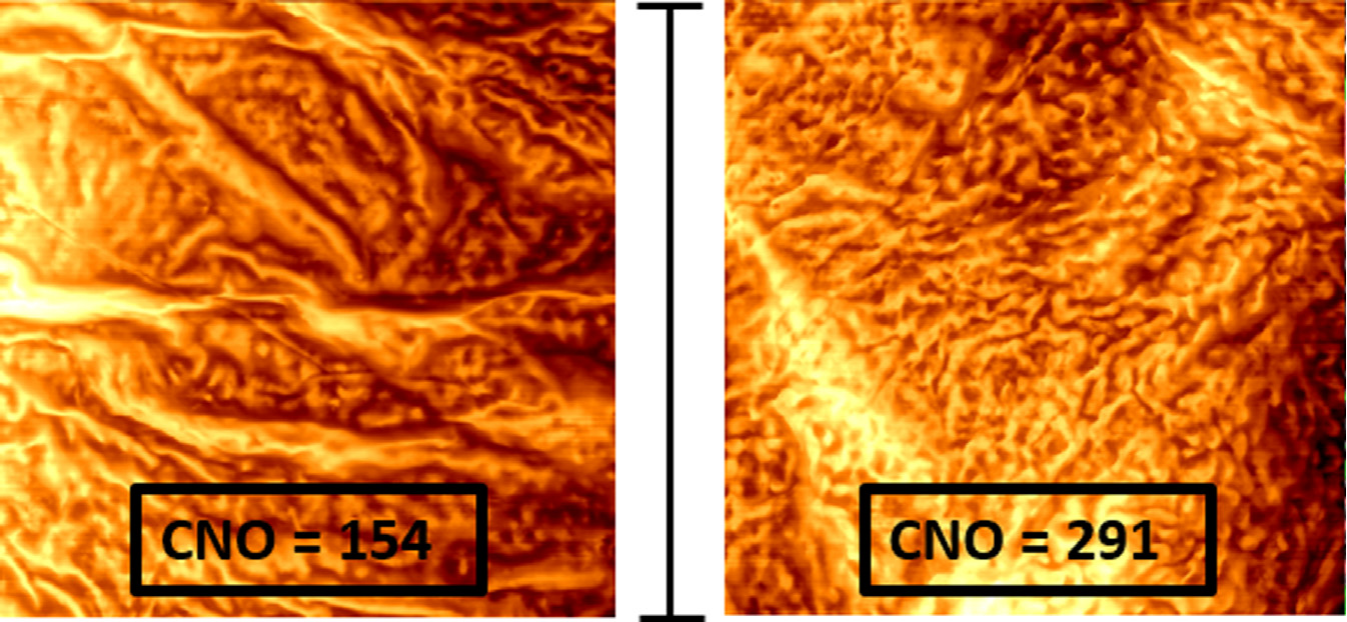
**Representative AFM images from 2 skin sites with high and low numbers of CNOs (number of CNOs/400 μm**^**2**^**).** From less sun-exposed skin site E (left) and more sun-exposed skin site F (right), measured with AFM. Bar = 20 μm. E denotes retroauricular skin, and F denotes forehead skin. AFM, atomic force microscopy; CNO, circular nano object.

The number of CNOs on the corneocyte surface, indicative of skin barrier, was significantly higher in the forehead skin than in the retroauricular skin location (Figure 1 and Table 2). Representative AFM images in Figure 2 illustrate corneocyte surface topography from sites with varying CNO counts.

Both the relative amount of cis-UCA and the total amount of UCA (sum of the cis-UCA and *trans*-UCA) were significantly higher in the forehead skin.

### Comparing 2 occupational groups (IWs vs OWs)

Among the 13 assessed biomarkers, significant differences were observed in the relative amount of cis-UCA and CCL4 between the 2 occupational groups (Figure 1 and Table 2). Both cis-UCA and CCL4 were notably higher in the OW group.

## Discussion

This study explored differences in SC biomarkers between 2 skin locations and 2 occupational groups with contrasting expected solar UVR exposure: the forehead and retroauricular skin sites as well as between IWs and OWs.

Our initial assumption that both forehead skin and OWs have higher UVR exposure than the corresponding retroauricular skin and IWs is supported by the higher relative amount of cis-UCA in the forehead skin and among OWs. Unlike its *trans*-isomer UCA, cis-UCA is not endogenously present in the skin but is formed upon exposure to UVB radiation until it reaches a photostationary state, typically at approximately 60–70% of the total UCA. Photoisomerization of *trans*-isomer UCA is a physical reaction, making cis-UCA a highly specific marker of UVR exposure (Bernard et al, 2019; Kammeyer et al, 1997; Landeck et al, 2016; Ruegemer et al, 2002; van der Molen et al, 2000; Vieyra-Garcia and Wolf, 2018). In addition to its role as an exposure biomarker, cis-UCA can be regarded as an effect biomarker owing to its immunomodulatory properties (Bernard et al, 2019).

The difference in SC biomarker levels was more pronounced between the 2 skin locations—forehead and retroauricular skin—than between IWs and OWs, consistent with the higher contrast in relative cis-UCA observed between these body locations than between IWs and OWs. Lower values for IL-1α were found in the forehead skin, whereas IL-1RA, IL-18, and IL1RA/IL-1α were higher. These findings align with results reported in experimental UVB-exposure studies in human volunteers (Keurentjes et al, 2022, 2020b). In addition to IL-1 cytokines, we also observed significantly higher levels of CCL4 in the forehead skin. CCL4, also known as MIP-1β, is a cytokine involved in attraction of leukocytes toward inflamed skin sites. Interestingly, Mai et al (2021) reported an inversed association between blood CCL4 levels and solar UVR exposure in a population-based study.

In our study, we included 15-HETE as a potential UVR biomarker, which had not previously been determined in SC tape strips. The 15-HETE is an anti-inflammatory eicosanoid that may temper the proinflammatory milieu in sunburn (Nicolaou et al, 2012). We observed significantly higher levels of 15-HETE in skin site forehead than of retroauricular skin. Previous research has shown that FLG-deficient epidermis is more susceptible to UV damage (Mildner et al, 2010) and contains higher levels of 15-HETE than FLG-sufficient epidermis (Blunder et al, 2017). Given the anti-inflammatory properties of 15-HETE (Nicolaou et al, 2012), it may be hypothesized that the elevated 15-HETE levels may act as a compensatory mechanism to protect against UV damage.

Next to immunological biomarkers, we explored corneocyte surface topography as a marker of the skin barrier. Prolonged exposure to sunlight can induce changes in the mechanical and structural properties of the SC (Biniek et al, 2012). Our findings demonstrated an increased presence of CNO in the more exposed forehead skin site. CNO has previously been suggested as an indicator of corneocyte maturation, which is crucial for cellular cohesion and skin barrier function (Riethmüller, 2018). Moreover, CNO has been proposed as a biomarker for UVR-related actinic damage in patients with actinic keratosis (Keurentjes et al, 2020a). Another skin barrier biomarker that was investigated in this study was total UCA, quantified as the combined sum of cis- and *trans*-isomer. UCA plays an important role in skin barrier function by maintaining the hydration and acidity of the SC (Thyssen and Kezic, 2014). The mayor source of UCA in the SC is the epidermal protein FLG (Thyssen and Kezic, 2014). In this study, we found higher levels of total UCA in the forehead skin than in the retroauricular skin. This increase might be caused either by UV-induced increase in the expression of FLG protein or elevated activity of proteases responsible for degradation of FLG into UCA.

One future application of SC biomarkers is to estimate internal UVR exposure in occupational settings and to enable assessing the effectiveness of interventions designed to reduce UVR exposure. In this study, we found a significant difference between IWs and OWs for the relative amount of cis-UCA and CCL4, which also showed a significant difference between the forehead and retroauricular skin. This finding suggests that CCL4 and relative amount of cis-UCA may be valuable biomarkers in detecting differences among groups of workers with varying levels of solar UV exposure, such as intervention and control group.

The study has several limitations. The contrast in UVR exposure between IWs and OWs, as indicated by the relatively small difference in relative cis-UCA, was modest. This might be attributed to the contribution of leisure solar exposure in both groups, resulting in a less distinct contrast between IWs and OWs. Our sample collection took place in September, after a very sunny period in The Netherlands. This is reflected in the relatively high relative cis-UCA values observed also in IWs. It is possible that in future studies involving intervention groups using sunscreen and control groups without sunscreen, with a more substantial contrast in sun exposure, immunological biomarkers may be more effective in detecting differences between these groups.

Regrettably, we lacked information about individual UVR exposure during both work and leisure, which could have provided a more comprehensive understanding of the relationship between UV dose and biomarker levels.

Another limitation of the study concerns the greater number of individuals with darker skin phototype in the OW group, potentially influencing the effects of UVR and, subsequently, biomarker levels.

SC biomarkers demonstrate promise in assessing the effects of chronic UVR exposure, encompassing both skin barrier and immunological markers. To explore their utility in evaluating the effectiveness of preventive measures aimed at reducing UVR exposure, larger cohort studies with solar exposure measurements are warranted.

## Materials and Methods

### Subjects

The applied definition of an outdoor worker was working outside for at least 4 hours during a working day. Exclusion criteria were age <18 years and female participants because most construction workers are men and because differences may be measured between the biomarkers in the skin of males and females (Rahrovan et al, 2018). Moreover, we excluded patients with visible skin conditions such as dermatitis or (solar) allergy, the intake of systemic immune suppressants or application of topical corticosteroids at the sampling site within 3 days prior to sampling, and the use of sunbeds or leisure excessive UVR exposure 1 month or less prior to collection of the skin samples. Informed consent was obtained from all participants prior to the study. The local Ethics Committee (Medisch Ethische Toetsingscommissie, Amsterdam University Medical Center, Amsterdam, The Netherlands) issued an exemption for this study.

### Sample collection

**Figure 3:**
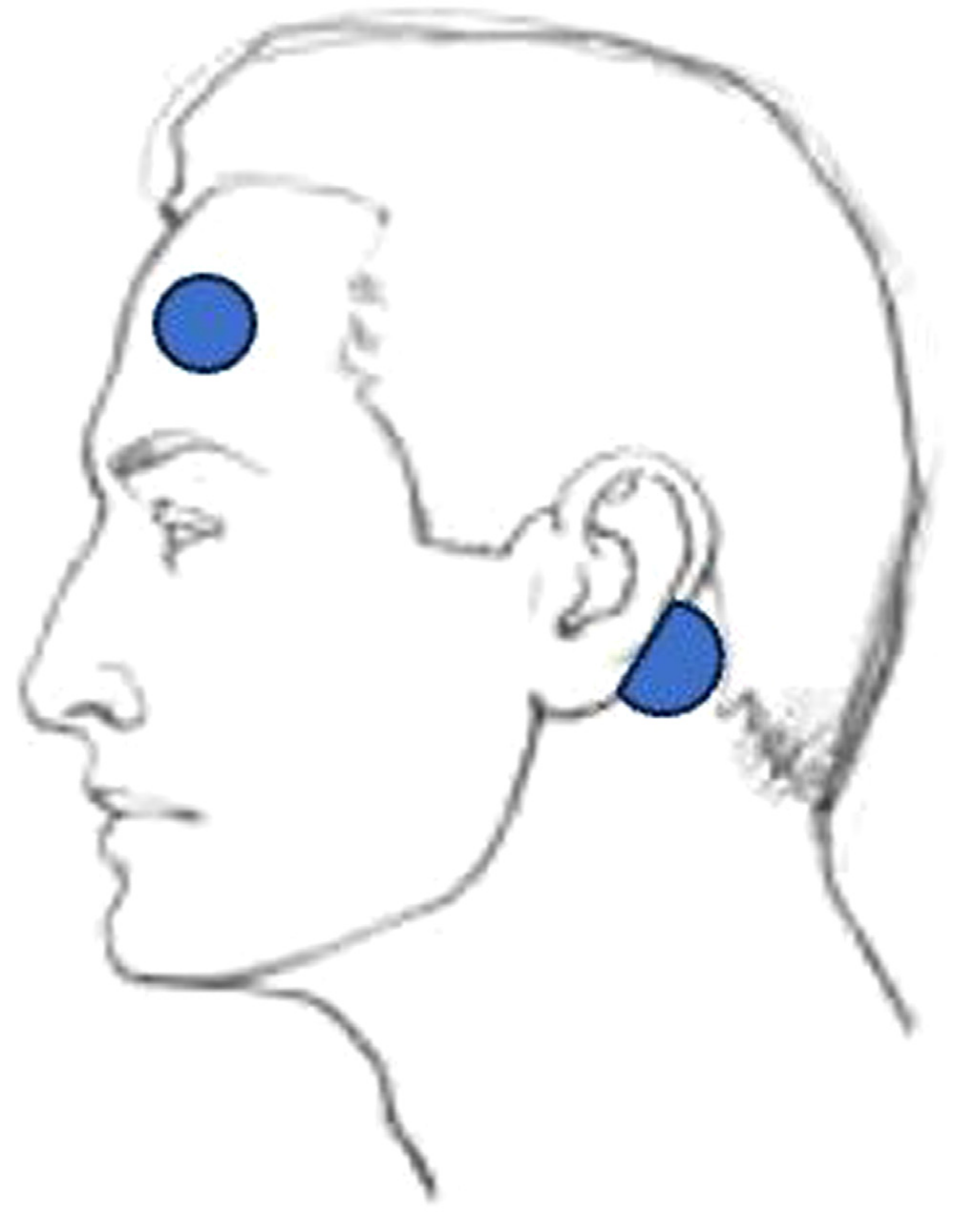
**Sampling sites: F and E skin locations.** E denotes retroauricular skin, and F denotes forehead skin.

Subjects were visited for collection of the skin samples at their workplace. During the visit, the outermost layer of the skin, the SC, was collected using adhesive tape strips by a method that is validated in experimental studies (Hulshof et al, 2019; Riethmüller, 2018). Briefly, round adhesive tape discs (3.8 cm^2^, DSquame, CuDerm) were attached to the skin. Each tape was pressed on the skin for 5 seconds with standardized force, using a disc pressure applicator (CuDerm). Tape strips were gently removed with tweezers and placed in a sampling vial. From each skin site, 6 successive tapes were collected and stored at −80 °C. The 6 tape strips were analyzed on the following candidate biomarkers: the first tape strip was discarded, the second and fourth tapes were used for 15-HETE analysis, the third tape strip was used for AFM (CNO), the fifth was used for immunological markers, and the sixth was used for UCA analysis. The tape strips were collected from the forehead and retroauricular skin (Figure 3).

### Biomarker analysis

#### The 15-HETE

The 15-HETE was extracted from tapes by adding 240 μl sample diluent from the assay kit to collecting vial and then transferred to UV-safe vials. Samples were subsequently thoroughly vortexed for 1 minute at 4 °C and placed on a rolling device for 5 additional minutes. The rolling device is an instrument for mixing liquid in a falcon tube, very suitable for antibody hydridization in western blotting and for impregnating tissue samples. In addition, liquids can be mixed gently without much froth in falcon tube. Then, samples were further vortexed for 15 seconds and shortly spanned down. ELISA immunoassay (Abcam) was carried out according to manufacturer’s instruction (Tan et al, 2020).

#### CNO

The third tape was glued onto glass slides and subjected to AFM contact imaging with no further preparation. In brief, 10 randomly chosen corneocyte areas of 20 μm were subjected to a new generation AFM imaging in contact mode. The approach involved using a silicon-nitride aluminum–coated AFM probe (spring constant of 0.03 N/m, CSC38/Al, MikroMasch) with a tip end radius of 8 nm. Each SC nanotexture image had a resolution of 512 × 512 pixels and imaging area of 20 × 20 μm. To ensure consistent measurement results, the contact forces between the AFM tip and the SC surface were kept below 10 nN. Ten areas on each SC sample were randomly selected to characterize the nanotexture. CNOs of size <500 nm were counted by a machine learning algorithm. The average count of 10 areas of (20 μm) 2 is referred to as Dermal Texture Index. AFM was performed on all skin sites (retroauricular and on the forehead) and in both groups (IWs and OWs).

#### Immunological markers in the SC

To the cryo-vial containing the 5th tape, 1.2 ml PBS (Merck, Darmstadt, Germany) with 0.005% Tween 20 (Sigma-Aldrich, Zwijndrecht, The Netherlands) was added. Extraction of immunological markers and soluble proteins was performed using an ultrasound bath (Branson 5800, Branson Ultrasonics, Ede, The Netherlands) for 15 minutes in ice water. Extract aliquots of 200 μl were distributed in cryo-vials and stored at −80 °C until further analysis. In total, concentrations of 8 cytokines, chemokines, GFs, angiogenesis factors, and matrix metalloproteinases were determined using MESO QuickPlex SQ 120 (Meso Scale Diagnostics, Rockville, MA) according to the manufacturer’s instructions. The following markers, involved in UVR-response pathways, were included: IL-1α; IL-1RA; IL-1RA/IL-1α ratio, which is often used as an indicator of inflammatory status (Angelova-Fischer et al, 2012); IL-18; macrophage inflammatory protein CCL4 (MIP-1β); cutaneous T-cell–attracting chemokine CCL27 (CTACK); matrix metalloproteinase 9; basic vascular endothelial GFs (VEGF-A); and thymus- and activation-regulated chemokine CCL17 (TARC). Because the amount of the SC on the tape varies, the amount of immunological marker in the SC on each tape was normalized by the protein content, which was determined using the Pierce Micro BCA Protein Assay Kit (Thermo Fischer Scientific, Rockford, IL), with the BSA supplied as standard. For 15-HETE, the protein analysis could not be performed, so the absolute values are given. If, for a specific marker, the majority (>50%) of the samples were under the detection level, that cytokine was excluded from data analysis.

#### UCA isomers in the SC

The sixth tape strip from investigated skin sites was used to measure UCA isomers according to the slightly adopted method described in detail elsewhere (Dapic et al, 2013). Briefly, *trans*- and cis-UCA on the tape strip were extracted with 600 μl of ultraclean water and subsequently analyzed by high-performance liquid chromatography equipped with UV detector. UCA concentration was corrected for protein amount as described elsewhere (Jurakic Toncic et al., 2020; Van Gool et al, 2020). The relative amount of cis-UCA was calculated as cis-UCA/total UCA, as an indicator for UVR exposure and to correct for differences in the levels of *trans*-isomer UCA between subjects.

### Statistical analysis

Data analyses were performed with SPSS, version 28, and GraphPad Prism 9.5.1. A linear mixed model was created, and normality testing was performed. The model used a logarithmic model and tested for the effects of occupational setting (IWs/OWs) and the effects of skin location (forehead/retroauricular skin) as well as the interaction between the 2 on the logarithm of the skin biomarker concentrations. The linear mixed model were adjusted for age and skin phototypes and included a random intercept for each individual. By means of a 2-stage step-up procedure of Benjamini–Hochberg false discovery rate test, we corrected for multiple testing (Benjamini and Hochberg, 1995). Outliers were identified and excluded from data analysis using the ROUT method (Motulsky and Brown, 2006).

## Ethics Statement

Informed consent was obtained from all participants prior to the study. The local Ethics Committee (Medisch Ethische Toetsingscommissie, Amsterdam University Medical Center, Amsterdam, The Netherlands) issued an exemption for this study.

## Data Availability Statement

This paper does not include large-scale databases. However, all data generated during and/or analyzed during these studies are available from the corresponding author on reasonable request.

## ORCIDs

Florentine L. de Boer: http://orcid.org/0009-0003-7595-7608

Sanja Kezic: http://orcid.org/0000-0002-1063-4547

Henk F. van der Molen: http://orcid.org/0000-0002-0719-2020

Edwin En-Te Hwu: http://orcid.org/0000-0002-5971-4978

Ivone Jakasa: http://orcid.org/0000-0002-7961-4069

Sandrine Dubrac: http://orcid.org/0000-0002-2936-8488

Thomas Rustemeyer: http://orcid.org/0000-0001-7580-0684

Jen-Hung Wang: http://orcid.org/0000-0001-9214-5837

Ellen Raun: http://orcid.org/0009-0007-6403-812

## Conflict of Interest

The authors state no conflict of interest.
